# Supplementation of multi-enzymes alone or combined with inactivated *Lactobacillus* benefits growth performance and gut microbiota in broilers fed wheat diets

**DOI:** 10.3389/fmicb.2022.927932

**Published:** 2022-08-01

**Authors:** Qingtao Gao, Yanchun Wang, Jiaheng Li, Guosong Bai, Lei Liu, Ruqing Zhong, Teng Ma, Hongbin Pan, Hongfu Zhang

**Affiliations:** ^1^State Key Laboratory of Animal Nutrition, Institute of Animal Science, Chinese Academy of Agricultural Sciences, Beijing, China; ^2^Yunnan Provincial Key Laboratory of Animal Nutrition and Feed Science, College of Animal Science and Technology, Yunnan Agricultural University, Kunming, China; ^3^Precision Livestock and Nutrition Unit, Gembloux Agro-Bio Tech, Liège University, Gembloux, Belgium

**Keywords:** multi-enzymes, inactivated *Lactobacillus*, growth performance, microbiota, broilers

## Abstract

The effects of multi-enzymes mixture supplementation or combination with inactivated *Lactobacillus* on growth performance, intestinal barrier, and cecal microbiota were investigated in broilers at the age of 15–42 days fed a wheat-based diet. A total of 576 broilers (12 broilers/cage; *n* = 12) were used and divided into four groups and randomly allotted to four experimental diets throughout grower (15–28 days of age) and finisher (29–42 days of age) phases. Diets consisted of a corn-soybean meal-based diet (BD), a wheat-soybean meal-based diet (WD), and WD supplemented multi-enzymes (WED) or combined with inactivated *Lactobacillus* (WEPD). The results showed that the average daily gain (ADG) and body weight (BW) were reduced in broilers fed WD diet compared with those fed BD diet during the grower period (*P* < 0.05). Broilers in the WED or WEPD group had higher ADG and BW during the grower period (*P* < 0.05) and had a lower feed-to-gain ratio (F/G) compared to broilers in the WD group during the grower and overall periods (*P* < 0.05). Improved expression of intestinal barrier genes (claudin-1, ZO-1, and mucin-2) was observed in WEPD compared to the BD or WD group (*P* < 0.05). Compared to the BD group, the WD group decreased the abundance of *Oscillospira*, *norank_f__Erysipelotrichaceae*, and *Peptococcus*, which are related to anti-inflammatory function and BW gain. The WD also increased *Bifidobacterium* and some short-chain fatty acid (SCFA)-producing bacteria (*Anaerotruncus*, *Blautia*, and *Oscillibacter*), and *Barnesiella*, which were presumed as “harmful microbes” [false discovery rate (FDR) < 0.05]. WED and WEPD groups, respectively, improved *Bilophila* and *Eubacterium_hallii_group* compared with those in the WD group (FDR < 0.05). In addition, the *Enterococcus* abundance was reduced in the WEPD group compared to the WD group (FDR < 0.05). Higher acetate and total SCFA concentrations were observed (*P* < 0.05) among broilers who received a WD diet. Compared with the WD group, the WED or WEPD group further increased cecal propionate content (*P* < 0.05) and tended to improve butyrate concentration. These results suggested that supplemental multi-enzymes alone and combined with inactivated *Lactobacillus* could improve the growth performance based on the wheat-based diet and offer additional protective effects on the intestinal barrier function of broilers.

## Introduction

Wheat is widely used in poultry diets across Europe, consisting of up to 70% by weight of the diet (Ball et al., 2013). However, non-starch polysaccharides (NSPs) present in wheat could reduce feed nutrient digestion and growth performance of broiler (Kiarie et al., 2014; Romero et al., 2014). The NSP, a kind of complex high molecular weight carbohydrate, can be categorized into water-soluble NSP or insoluble NSP (Musigwa et al., 2021). The NSP disturbs digestion and absorption by resisting the digestion of endogenous enzymes (Nguyen et al., 2021), increasing the viscosity of the intestinal contents (Knudsen, 2014; Munyaka et al., 2016), or blocking the nutrients’ access to endogenous digestive enzymes in poultry (Choct et al., 2004; Musigwa et al., 2021). Moreover, intestinal microbiota composition is impacted by NSP in wheat-based diets (Munyaka et al., 2016; Wang et al., 2021b), which further leads to growth performance reduction (Crisol-Martínez et al., 2017) and intestinal physiological disfunction (Raza et al., 2019). Crisol-Martínez et al. (2017) reported that several operational taxonomic units (OTUs) belonging to *Clostridiales* are prevalent in wheat-fed chickens and associated with low FCR values. Also, other studies found correlations between the cecal microbiota composition and growth performance in broilers fed wheat-based diets (Torok et al., 2011; Stanley et al., 2012).

Supplementation of NSP-degrading enzymes or probiotics has been commonly accepted to eliminate the possible antinutritional effects of wheat-based diets and improve production performance and intestinal health. Studies showed that an enzyme mixture addition in wheat diets improved nutrient digestibility and broiler performance, especially for high NSP concentration wheat (Adeola and Cowieson, 2011; Smeets et al., 2018). The beneficial mechanisms of NSPases include the release of nutrients and the reducing chyme viscosity through hydrolysis. However, McCafferty et al. (2019b) reported that no effects of supplementation of NSPases on growth performance and cecal short-chain fatty acids (SCFAs) concentrations were observed. Rebolé et al. (2010) also found that enzymes added to the diet have no effects on specific selected bacterial populations. These inconsistent observations could be ascribed to the specificity of NSPases for specific NSP or feed ingredients (Smeets et al., 2014) and the various quality of wheat (Choct et al., 1995). On the other hand, supplementation of probiotics also can improve the growth performance and regulate the balance of bacteria in broilers (Alagawany et al., 2018; Rehman et al., 2020). A previous study showed that diet addition inactivated *Lactobacillus* to broilers diet increased body weight (BW) gain and FCR, and hypertrophied intestinal absorptive epithelial cells are found at the villus apical surface (Khonyoung and Yamauchi, 2019). In agreement with that finding, Incharoen et al. (2019) observed that adding inactivated *Lactobacillus* improved the growth performance and immune function in broilers. Besides, in yellow-feathered broilers, diet supplementation with heat-inactivated compound probiotics (*Bacillus subtilis* and *Lactobacillus acidophilus* BFI) modulated the community composition of cecal microbiota, including the high abundance of *Barnesiellaceae*, *Barnesiella*, and *Lactobacillus aviaries*, whereas the low abundance of *Lachnoclostridium* and *Peptococcus*, consequently enhanced feed efficiency and decreased plasma cholesterol and creatinine contents (Zhu et al., 2020). At present, most studies focused on the effects of a single NSP enzyme or probiotics. However, there is a limited understanding of the combined effects of multi-enzymes and probiotics on growth performance, intestinal barrier function, and intestinal microbiota in broiler fed a wheat diet.

Therefore, this study investigated the effects of NSP enzyme mixture and combined with probiotics on growth performance, intestinal barrier, gut microbiota, and microbial metabolites in broilers fed wheat diet from 15 to 42 days of age.

## Materials and methods

### Enzyme mixture and probiotics

The kinds and amounts of multi-enzymes used in this study were screened and optimized by our previous study (Zhang et al., 2017). The multi-enzymes included cellulase, xylanase, β-mannanase, α-galactosidase, β-glucanase, and pectinase. The addition amount was 1,117.9, 35,087.7, 1,917.1, 305.0, 806.7, and 133.7 U/kg of diet, respectively. The probiotic was inactivated *Lactobacillus*, containing more than 1 × 10^10^ inactivated bacterial cells/g, and was supplemented to diet at a level of 800 mg/kg recommended by the company (Shandong Biocom Biotech Co., Ltd., Linyi, China), which has been investigated and obtained a good input-output ratio in our previous experiments (data unpublished).

### Birds, housing, and experimental diets

One-day-age Arbor Acres broilers were randomly assigned into 48 cages containing 12 broilers each. All broilers were fed a corn-based diet for the first 14 days. On day 15, a total of 576 chicks were used and individually weighed (406.2 ± 9.8 g), then divided into 4 groups with 12 replicates per group and 12 broilers per replicate, to evaluate the effects of the 4 different diets on broilers in a completely randomized design. Broilers were offered a grower diet from day 15 to 28, and a finisher ration from day 15 to 28. Birds were kept in three-layer pens (150 cm × 70 cm × 60 cm for one cell). Feed and water were supplied *ad libitum* with a light schedule of 23-h light and 1-h darkness per day throughout the entire experiment. The room temperature gradually decreased from 35 (d1–d3) to 24°C (d28). After 28 days of age, the temperature was kept at 22∼24°C until the end of the experiment.

The diets contained a corn-based diet (BD), wheat replacement diet (WD), and wheat replacement diet with an enzyme mixture alone or combined probiotics (WED or WEPD), resulting in four diet treatments for grower and finisher stages, respectively. All diets met or exceeded the nutrient requirements of broilers according to the National Research Council (1994) (Table 1). All diets were fed in mash form. No antibiotics were offered to broilers throughout the trial.

**TABLE 1:** Composition of the basal diets (%, as fed).

Item	Grower		Finisher	
	BD	WD	BD	WD
Corn	58.38		60.13	
Wheat		68.09		70.81
Soybean meal	32.05	21.28	29.47	17.88
Soybean oil	5.00	5.79	5.69	6.39
Limestone	1.10	1.10	1.20	1.10
Salt	0.30	0.30	0.30	0.30
CaHPO4	1.50	1.50	1.50	1.50
L-Lysine HCl	0.19	0.40	0.22	0.46
DL-methionine	0.28	0.25	0.29	0.26
L-threonine	0.10	0.19	0.10	0.20
Premix^1^	1.00	1.00	1.00	1.00
Choline chloride	0.10	0.10	0.10	0.10
Total	100.00	100.00	100.00	100.00
**Calculated nutrient levels, %**				
Calcium	0.82	0.89	0.85	0.89
Total phosphorus	0.60	0.65	0.59	0.64
Available P	0.37	0.37	0.37	0.37
Lys	1.01	1.01	0.98	0.98
Met	0.52	0.48	0.76	0.76
Thr	0.71	0.71	0.68	0.68
ME, Mcal/kg	3.07	3.07	3.12	3.12
**Analyzed value, %**				
DM	88.94	90.25	90.59	91.89
GE, Mcal/kg	4.12	4.20	4.58	4.65
CP	19.39	20.00	18.92	19.15

^1^Supplied per kilogram of diet: vitamin A, 15,000 IU; vitamin D3, 3,450, IU; vitamin E, 22.5 IU; vitamin K, 2.25 mg; thiamine (vitamin B1), 2.7 mg; riboflavin (vitamin B2), 8.4 mg; pyridoxine (vitamin B6), 4.86 mg; vitamin B12, 0.03 mg; niacin, 44.55 mg; folic acid, 1.47 mg; biotin, 0.18 mg; pantothenic acid, 16.56 mg; Cu, 8.5 mg; Fe, 102 mg; Zn, 72.76 mg; Mn, 97.34 mg; I, 0.48 mg and Se, 0.3 mg.

### Chemical analysis

All experimental diets were ground to pass through a 0.43-mm screen before analyses. Dry matter (method 934.01) and crude protein (nitrogen × 6.25, method 990.03) contents of diets were determined according to the AOAC (2012). Gross energy contents in the diet samples were analyzed using an adiabatic oxygen bomb calorimeter (model 6400, Parr Instruments, Moline, IL, United States).

### Growth performance and sample collection

Birds and their feed in each replicate cage were weighted and recorded at the beginning and end of each period (d15, d28, and d42), respectively, to calculate average BW, average daily gain (ADG), and average daily feed intake (ADFI) of each treatment during the grower (15–28 days), finisher (29–42 days), and overall (15-42 days) periods. The ratio between ADFI and ADG was also calculated (F/G).

At the end of the experiment, chickens were selected for intestinal sample collection. One bird from each cage was chosen according to the BW which was closed to the average, with a total of eight individuals from each treatment. The jejunal mucosa and cecal digesta were collected and frozen at –80°C for further analysis.

### Inflammatory cytokines and tight junction gene expression

Total RNA of the jejunal mucosa was isolated using the RNeasy Kit (Qiagen, Hilden, Germany) according to the manufacturer’s directions. The reverse transcription was completed using a Prime Script RT Kit (Takara, Kusatsu, Shiga, Japan). The quantitative real-time-PCR was conducted on a CFX96 real-time PCR detection system (Bio-Rad, CA, United States) according to the SYBR Premix Ex Taq II recommendations (Takara, Kusatsu, Shiga, Japan). The reaction protocol was 30 s for predenaturation at 95°C, 5 s for 40 cycles of denaturation at 95°C, 30 s for annealing at 60?, and 10 s for an extension at 95?, then melt curve analysis was done. All primer sequences for genes associated with immune function and intestinal barrier function are provided in Supplementary Table 1. β-Actin was used as an internal reference gene, and the relative gene expression was calculated by the 2^–ΔΔ*Ct*^ *^Ct^* method (Livak and Schmittgen, 2001).

### Intestinal microbiota

The extraction of DNA of cecal digesta samples was performed using the Qiagen DNA isolation kit (Qiagen, Hilden, Germany), following the instructions of the manufacturers. The V3–V4 region of the bacterial 16S rRNA gene was amplified with primers 338F (5′-barcodeACTCCTACGGGAGGCAGCAG-3′) and 806R (5′-GGACTACHVGGGTWTCTAAT-3′). The purified amplicons were paired-end sequenced (2 × 300) on the Illumina HiSeq sequencing platform (Illumina, San Diego, CA, United States) according to the standard protocols by Majorbio Bio-Pharm Technology Co. Ltd. (Shanghai, China), as previously described (Tang et al., 2021a).

Raw fastq files were demultiplexed and quality-filtered by QIIME (version 1.70). OTUs were clustered using a 97% similarity cut-off with UPARSE (version 7.1), and chimeric sequences were removed using UCHIME (version 7.1). OUT representative sequence was obtained based on the RDP classifier with a confidence threshold of 0.7 (Wang et al., 2007).

### Short-chain fatty acid concentration

The short-chain fatty acid determination in the caecum digesta was performed according to the procedure described previously (Tang et al., 2021b). In brief, the digesta (about 0.5 g) was extracted with ultrapure water, and centrifugation at 10,000 × *g* to obtain the extracts. Metaphosphoric acid (25%, w/v) was mixed with the extracts at a ratio of 1:9. After centrifugation at 12,000 × *g*, the supernatant was passed through the 0.45-μm Milled-LG filter (Millipore, Billerica, MA, United States) and subjected to SCFA analysis with the Agilent 7890 N gas chromatograph (Agilent, Santa Clara, CA, United States).

### Statistical analysis

Data on growth performance, carcass traits, and intestinal morphology were analyzed by one-way ANOVA with Duncan’s multiple comparison test to determine differences among treatments using SAS 8.0 (SAS Institute, Inc., Cary, NC, United States). The data from a replicate cage were calculated as the experimental unit for the growth performance evaluation, involving BW and feed intake. The bird individual from each cage was regarded as the basic unit for physiological and microbial analysis.

Microbial analysis was performed using the online platform of Majorbio I-Sanger Cloud Platform.^1^ Bacterial alpha-diversity indices (Sobs, Shannon, ACE, and Chao) were analyzed using Wilcoxon rank-sum test, and the principal coordinate (PCoA, beta-diversity) analysis based on the unweighted unifrac and ANOSIM test was employed. Also, a partial least squares discriminant analysis was performed to predict the group to which the samples belonged, according to the diet. After the OUT data were employed in the CSS normalization, the analysis of differential genera between groups was calculated by the DESeq2 method (MicrobiomeAnalyst^2^) with corrected *p*-value [false discovery rate (FDR)] < 0.05.

## Results

### Growth performance

In Table 2, the initial BW at 14 days was not different among the four groups (*P* = 0.92). Birds fed the WD diet showed lower ADG and BW by 28 days compared with those fed the BD diet (*P* < 0.05). Whereas, the WED group or the WEPD group improved the ADG and BW (*P* < 0.05) compared to the WD group (*P* < 0.05), which was similar to the BD group. The WED group decreased ADFI (*P* < 0.05) but did not affect BW at 42 days (*P* > 0.05). In addition, compared to the WD group, WED and WEPD groups decreased the F/G during the grower and overall periods (*P* < 0.05).

**TABLE 2:** Effects of wheat diet supplement with enzyme mixture alone or combined with probiotics on broiler growth performance.^1^

Item	BD	WD	WED	WEPD	SEM	*P*-value
**Average body weight (g/bird)**						
At 14 days	407.5	404.8	406.2	407.5	3.15	0.92
At 28 days	1,260.6^a^	1,186.1^b^	1,267.8^a^	1,225.2^ab^	18.56	0.01
At 42 days	2,163.9	2,089.5	2,123.6	2,167.8	39.65	0.46
**Average daily feed intake (g/d)**						
D15-28	89.4	88.0	89.7	88.5	1.14	0.7
D29-42	125.1^a^	127.1^a^	115.6^b^	126.1^a^	3.05	0.04
D15-42	106.5	107.0	102.3	106.8	1.70	0.18
**Average daily gain (g/d)**						
D15-28	65.6^a^	60.1^b^	66.3^a^	62.9^ab^	1.33	0.01
D29-42	64.5	65.2	61.1	68.7	2.14	0.13
D15-42	65.1	62.9	63.6	65.2	1.40	0.6
**Feed to gain ratio**						
D15-28	1.47^bc^	1.58^a^	1.46^c^	1.52^b^	0.02	<0.01
D29-42	1.95	1.96	1.90	1.84	0.04	0.1
D15-42	1.70^b^	1.77^a^	1.67^b^	1.70^b^	0.02	0.02

^1^Each value represents the mean of 12 replicates. BD, basal diet; WD, wheat diet; ED, wheat diet supplemented with 300 mg/kg enzyme mixture; HPE, wheat diet supplemented with 300 mg/kg enzyme mixture and 100 mg/kg probiotics. Different letters in the same row indicate significant differences between the respective means (P < 0.05).

### Inflammatory cytokines and tight junction gene expression

**FIGURE 1:**
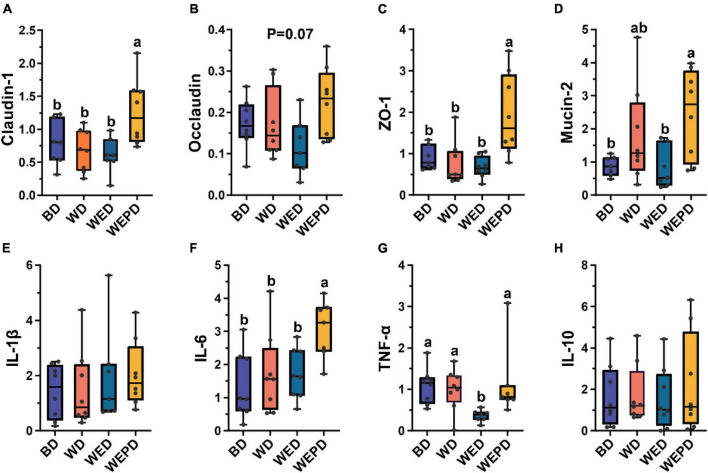
Expression of inflammatory cytokines and tight junction protein in the jejunal mucosa among different treatments. **(A–D)** mRNA expression of claudin, occludin, ZO-1, and mucin-2. **(E–H)** mRNA expression of IL-1β, IL-6, TNF-α, and IL-10. Data are expressed as min to max showing all points (*n* = 7∼8 broilers/group). Different letters (a, b) means significant differences (*P* < 0.05).

The wheat-soybean meal-based diet group increased mRNA expression of IL-6 and decreased major tight junction protein expression (claudin, occludin, and ZO-1) in the jejunum of broilers compared to the BD group, even though these did not reach the significant level (*P* > 0.05, Figures 1A–C,F). The WED group improved the expression level of TNF-α (*P* < 0.05, Figure 1G). In addition, the WEPD group showed higher mRNA expressions of jejunal mucosal claudin-1, ZO-1, and mucin-2 (*P* < 0.05, Figures 1A–D), and tended to increase the occludin expression (*P* = 0.07, Figure 1B). However, the WEP group also increased the expression level of IL-6 (*P* < 0.05, Figure 1F). There was no significant difference in expression of IL-1β and IL-10 among groups (*P* > 0.05, Figures 1E,H).

### Evaluation of sequencing data

A total of 1,536,879 valid sequences from 32 caecum samples were obtained (with sequences ranging from 34,325 to 61,141 per sample) after denoising and cleaning. The reads of all the samples were normalized based on the minimum number of sample sequences. A total of 34,325 reads in each sample was clustered into 645 OTUs, 163 genera, 72 families, 46 orders, 20 classes, and 11 phyla. Good coverage (>0.998) and rarefaction curves indicated that the sampling of each group provided sufficient sequences to reflect the microbial diversity and bacterial communities in cecum digesta samples (Supplementary Figure 1).

### Diversity and structure of intestinal microbiota

**FIGURE 2:**
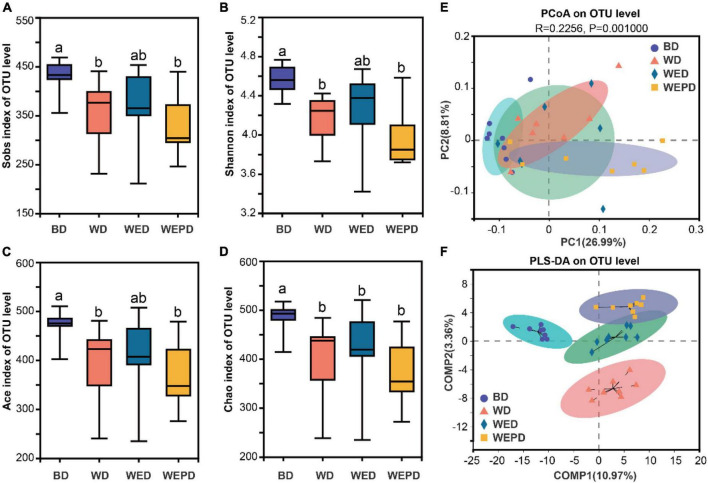
Alpha and beta diversities of the microbial community in the cecum digesta among different treatments. **(A)** The Sobs, **(B)** Shannon, **(C)** ACE, and **(D)** Chao indexes in the cecum among four groups; PCoA **(E)** and PLS-DA **(F)** of microbial community based on unweighted UniFrac and ANOSIM test in cecum digesta. Different letters (a, b) means significant differences (*P* < 0.05).

In the BD group, Sobs, Shannon, Ace, and Chao indexes were significantly higher than those in the WD and WEPD groups (Figures 2A–D; *P* < 0.05). The WED group tended to increase the α-diversity compared to the WD group and up to the level of those in the BD group (*P* > 0.05). The result of PCoA indicated that the microbial composition of broilers in the BD group separated from those in the other three diet groups (Figure 2E; *R* = 0.1693, *P* = 0.0020). No distinct clustering was observed among WD, WED, and WEPD groups. The PLS-DA showed distinct clusters among the four groups (Figure 2F).

### Composition and alteration of specific microbiota

**FIGURE 3:**
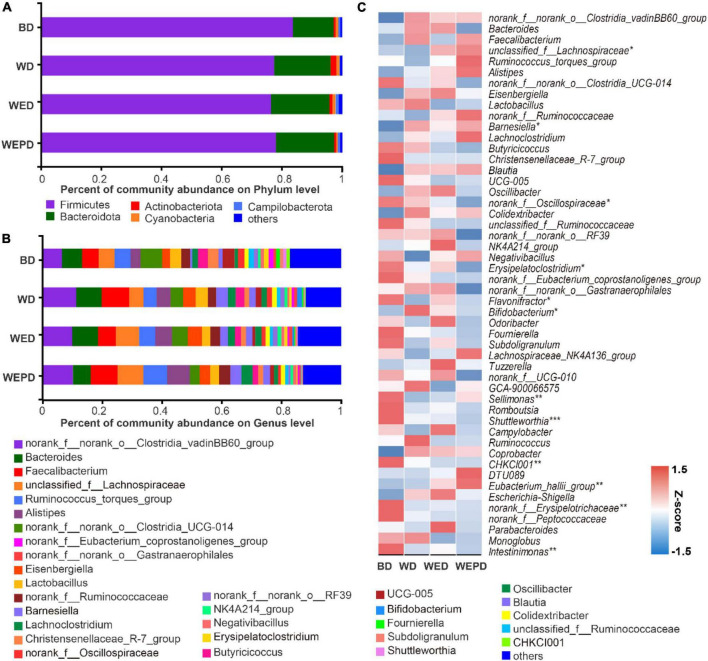
Community composition analysis. Phylum **(A)** and genus **(B)** level relative abundance, and trends change **(C)** of microbiota (phylum level) from the cecal digesta of broilers fed different diets.

At the phylum level (Figure 3A), the most abundant phyla were Firmicutes and Bacteroidetes, accounting for 97.07% on average in the BD group, 95.76% in the WED group, and 97.34% in the WEPD group (*n* = 8), respectively. The WD group, the WED group, and the WEPD group decreased the Firmicutes abundance and increased the Bacteroidota abundance, but none of them reached a significant level (*P* > 0.05).

At the genus level (Figure 3B), the top 10 dominant genera were *norank_f__norank_o__Clostridia_vadinBB60_group*, *Bacteroides*, *Faecalibacterium*, *unclassified_f__Lachnospiraceae*, *Ruminococcus_torques_group*, *Alistipes*, *norank_f__norank_o__Clostridia_UCG-014*, *Eisenbergiella, Lactobacillus*, *norank_f__Ruminococcaceae*, *Barnesiella*, and *Lachnoclostridium*, which consist of 52∼70% of total genera. The trends change in the top 50 genera of the cecal digesta attracted more attention and were presented in a heatmap (Figure 3C). The relative abundance of some genera notably decreased in broilers fed WD group compared to the BD group (e.g., *Flavonifractor*, *Subdoligranulum*, *Lachnospiraceae_NK4A136_group*, *Sellimonas*, and *Intestinimonas*; Figure 3C), but restored in the WED group. Other genera had no difference between BD and WD groups, but showed a remarkable variation in WEDP (e.g., *unclassified_f__Lachnospiraceae*, *norank_f__Oscillospiraceae*, *Erysipelatoclostridium*, *Eubacterium_hallii_group*, and *norank_f__Peptococcaceae*; Figure 3C).

**FIGURE 4:**
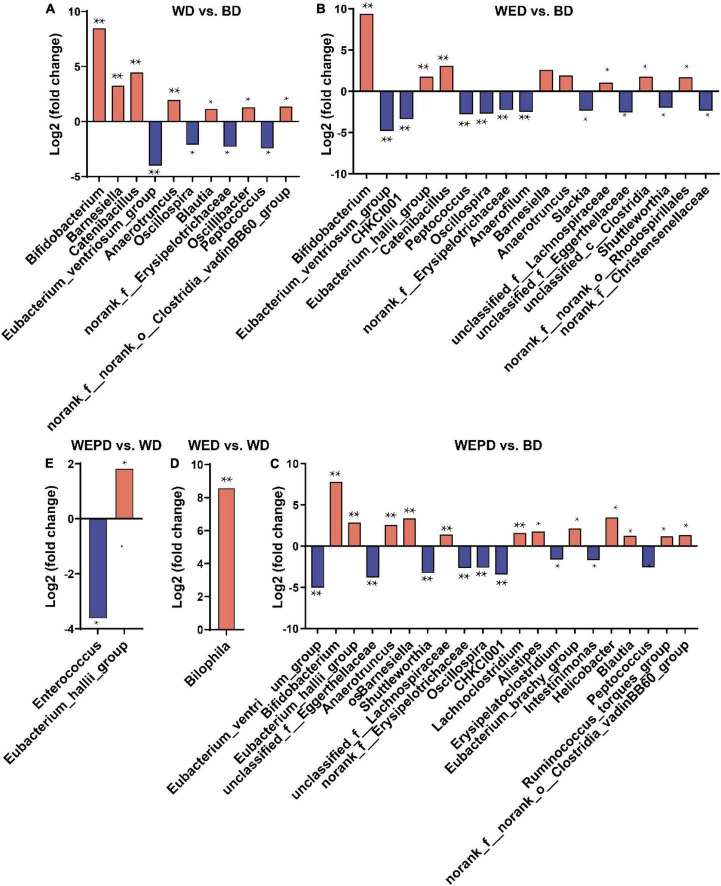
Differential genera analysis of cecum microbiota. **(A)** Pairwise comparisons between WD and BD groups using the DESeq2 method. Positive fold changes mean the genus is enriched in the WD group, while negative fold changes mean those enriched in the BD group. **(B)** Pairwise comparisons between WED and BD groups. **(C)** Pairwise comparisons between WEPD and BD groups. **(D)** Pairwise comparisons between WED and WD groups. **(E)** Pairwise comparisons between WEPD and WD groups. *P-value (FDR) < 0.05; **P-value (FDR) < 0.01.

Compared with the BD group, seven genera (*Bifidobacterium*, *Barnesiella*, *Catenibacillus*, *Anaerotruncus*, *Blautia*, *norank_f__norank_o__Clostridia_vadinBB60_group*, and *Oscillibacter*) were increased, while four genera (*Eubacterium_ventriosum_group*, *Oscillospira*, *norank_f__Erysipelotrichaceae*, and *Peptococcus*) were decreased in the WD group (FDR < 0.05, Figure 4A). The relative abundance of *Bilophila* in the WED group was increased compared with that in the WD group (FDR < 0.05, Figure 4D). The relative abundance of *Eubacterium_hallii_group* was improved, while the *Enterococcus* abundance was reduced in the WEPD group compared to the WD group (FDR < 0.05, Figure 4E). Beyond that, more differential genera were observed in WED and WEPD groups compared the BD group. The genera of *Eubacterium_hallii_group* and *unclassified_f__Lachnospiraceae* were enriched in WED and WEPD groups, whereas the genera of *Shuttleworthia*, *norank_f__Erysipelotrichaceae*, *CHKCI001*, and *Intestinimonas* were enriched in the BD group (FDR < 0.05, Figures 4B,C).

### Short-chain fatty acid concentration

**FIGURE 5:**
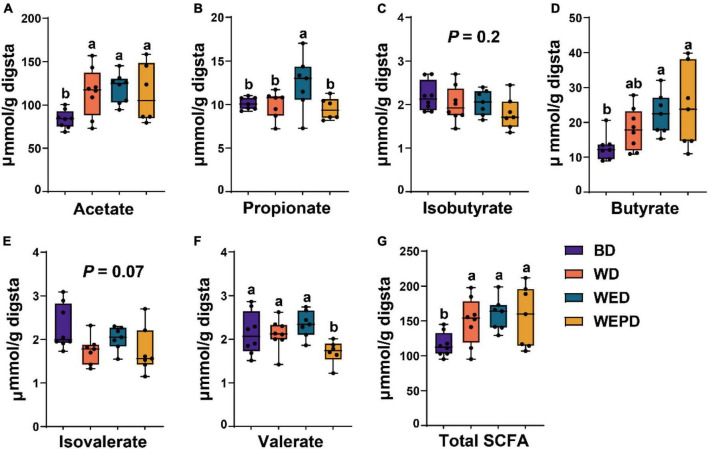
Concentrations of short-chain fatty acids. Difference of acetate **(A)**, propionate **(B)**, isobutyrate **(C)**, butyrate **(D)**, isovalerate **(E)**, valerate **(F)**, and total SCFA **(G)** among four diet treatments. Different letters (a, b) means significant differences (*P* < 0.05).

Short-chain fatty acids concentrations among different diet groups were shown in Figure 5. There was no difference in concentrations of propionate, isobutyrate, butyrate, isovalerate, and valerate in the WD and BD groups. Broilers receiving the WED diet improved (*P* < 0.05) propionate production, compared with the WD diet. Besides, WED and WEPD groups improved (*P* < 0.05) butyrate yield, compared with the BD group. The WEPD group had the lowest (*P* < 0.05) valerate concentration among all diet groups.

## Discussion

Supplementation of exogenous NSP enzyme or probiotics has been reported to improve growth performance and intestinal health of broilers fed wheat-based diets (De Maesschalck et al., 2015; Bhogoju et al., 2021; Wang et al., 2021a,b). The chemical structure and composition of NSP are varied and complex (Ward, 2021). Therefore, combining multiple NSPases that target different NSPs may result in greater benefits than using individual NSP-degrading enzymes (Munyaka et al., 2016). The use of probiotics improved balanced microbiota and good intestinal health, leading to better nutrient digestion and absorption (Alagawany et al., 2018), whereas, the effects of probiotic supplementation might not be consistent (Rehman et al., 2020). Moreover, the increasing use of probiotics may spread live bacteria to the environment (Kareem et al., 2017). Heat-inactivated *Lactobacillus* has been reported to stimulate the immune system and growth performance (Incharoen et al., 2019). However, there is a lack of information regarding the effects of multi-enzymes and combined with inactivated *Lactobacillus* on growth performance, intestinal barrier, and intestinal microbiota in broilers fed a wheat diet. The present results underscore the potential of the multi-enzymes mixture and combined with inactivated *Lactobacillus* to improve growth performance might be related to the microbial composition, metabolites, and intestinal barrier function in broilers.

In the current study, broilers fed a wheat-based diet showed lower ADG and higher F/G than broilers received a corn-based diet, which was consistent with published reports that feeding broilers with a wheat-based diet decreased BW gain from day 1 to 42 (Wang et al., 2021a) and BW at day 28 (McCafferty et al., 2019a), compared with that broilers fed a corn-based diet. Masey-O’Neill et al. (2014) also observed broilers fed a corn-based diet increased BW gain and feed intake and decreased F/G of broilers from day 1 to 28 and day 1 to 40 when compared with those fed a wheat-based diet. This might be related to the high concentration of soluble NSP in wheat, leading to the increase of intestinal digesta viscosity, affecting gut transit time, and thus reducing absorption and utilization of nutrients in broilers (Knudsen, 2014; Munyaka et al., 2016). However, McCafferty et al. (2019b) reported no effects of supplementation of NSPases on growth performance. These inconsistent observations could be ascribed to the specificity of NSPases for specific NSP or feed ingredients (Smeets et al., 2014) and the various quality of wheat (Choct et al., 1995). In the current study, supplementation of multi-enzymes alone or combined with inactivated *Lactobacillus* improved the growth performance of broilers, indicated by high ADG from day 14 to 28, high BW at day 28, and low F/G for the whole trial period. Similar to our results, it has been reported that broilers fed a wheat-based diet supplemented with enzymes or probiotics could improve growth performance (Zhao et al., 2016; Wang et al., 2021b). Khonyoung and Yamauchi (2019) also found that dietary supplementation with inactivated *Lactobacillus* increased body weight gain and FCR of broilers, which might be related to the reduction of digesta viscosity and protection of intestinal health, including intestinal immunity and intestinal barrier function improvement (Gonzalez-Ortiz et al., 2017; Bhogoju et al., 2021).

A healthy gut is extremely important, as the gut is related to the absorption and metabolism of nutrients and barrier function (Taciak et al., 2015; Xiong et al., 2019). The intestinal epithelial barrier function comprises physical, ecological, and immunological barriers, which positively affect nutrient digestion and utilization. In this study, the WD group tended to increase mRNA expression of IL-6 and decrease expression of occludin in the jejunum of broilers compared to the BD group. The IL-6 is an important proinflammatory cytokine that is considered to regulate pathological responses (Umare et al., 2014). The occludin is one of the tight junction proteins that is associated with modulating intestinal barrier function (Wang et al., 2020). The results suggested wheat diet impacted intestinal barrier function, which may lead to the decrease of ADG and FCR in broilers. Wheat diet supplemented enzymes inhibited the expression level of tumor necrosis factor (TNF)-α. In addition, supplementation of wheat diet with enzymes and probiotics improved mRNA expressions of jejunal mucosal claudin-1, ZO-1, and tended to improve mucin-2 and occludin expression. Similar to IL-6, TNF-α is another important proinflammatory cytokine. Claudin-1, ZO-1, and mucin-2 are also essential components of barrier function (Tornavaca et al., 2015). In previous studies, Wang et al. (2021b) found that supplementing xylanase in the wheat diet reduced IL-1β and TNF-α concentrations of ileal tissues in broilers, but no significant differences were observed in occluding and ZO-1 mRNA expression. However, some researchers focused on the exogenous enzyme complex reported a significant mRNA expression increase in claudin, occludin, and ZO-1, and also a decrease of IL-1β and TNF-α levels in the jejunum of nursery pigs and broiler, respectively (Tiwari et al., 2018; Wang et al., 2021c). Our results indicate that NSP enzymes or enzymes combined with probiotic supplementation could enhance intestinal barrier function. The healthy intestinal barrier function is beneficial to the effective digestion and absorption of nutrients in broilers, thus improving the growth performance of broilers. A large number of microorganisms inhabit the gastrointestinal tract, which is generally considered a “microbial organ.” The stabilization of the “microbial organ” is critical to gut health, barrier function, and nutrient uptake (Fraumene et al., 2018), contributing to better growth performance. Therefore, further analysis of the community structure of microbiota was performed. We found that the WD group had a lower alpha-diversity and separated from the BD group. It was consistent with a previous study that the corn-based diet increased the alpha-diversity of the cecal bacterial community and separated from the wheat-based diet (Wang et al., 2021a). A previous study reported that an increased α-diversity may promote the stability of microbiota, which is beneficial to health (Díaz Carrasco et al., 2018). The α-diversity was not affected by either NSP enzyme supplementing or combined with inactivated *Lactobacillus* in a wheat-based diet, which was also revealed by xylanase supplementation of wheat-based and corn-based diets in broilers (Wang et al., 2021a). Zhang et al. (2021) also found that *Bacillus subtilis* addition in the corn-based diet has no effects on the alpha diversity of microbiota in the cecum of broilers. Moreover, there was no distinct cluster among the WD group, the WED group, and the WEPD group, which was also similar to the results that no distinct clustering between the wheat diet group and the wheat diet added with the xylanase group was observed (Wang et al., 2021a). Those results suggested that the microbial community structure of broilers was mainly affected by types of diet rather than enzymes or probiotic supplementation.

The variations in the relative abundance of microbiota were further analyzed to illustrate the modulation of microbiota by supplemental enzymes and probiotics. We found that enzymes and probiotics modulated the abundance of specific bacteria without changing the overall microbial structure. In the current study, the abundance of *Bifidobacterium*, classified as Actinobacteria, was higher in the WD group compared with those in the BD group regardless of whether enzymes and probiotics were added. Consistent with the present study, Wang et al. (2021a) also reported that broilers fed a wheat-based diet has a high abundance of *Bifidobacterium* in the cecum. A previous study indicates that *Bifidobacterium* could produce the SCFA acetate (Engevik et al., 2021) by synthesizing exopolysaccharides as substrate (Salazar et al., 2008). This also explains the higher concentrations of acetate and total SCFA in the cecum of broilers fed wheat diet than those fed the BD diet. However, *Barnesiella*, presumed as “harmful microbes” (Han et al., 2021), was higher in the WD group compared to the BD group. The abundance of *Barnesiella* in the multi-enzyme supplementation group (WED) was reduced to the same levels as that in the BD group. Moreover, the genera *Blautia*, belonging to Firmicutes, was negatively correlated with the BW of the host (Ozato et al., 2019). Our current results demonstrated that broilers fed the WD diet had higher *Blautia* abundance in the cecum, which may be ascribed to the poor growth performance of broilers. Supplementation of NSP enzymes has improved growth performance and may be related to the decrement of *Blautia* abundance. *Blautia* is also well-known as the butyric-acid-producing and acetic-acid-producing bacteria in the gut microbiota (Kim et al., 2021). In addition, genera *Anaerotruncus* and *Oscillibacter* are also butyrate-producing bacteria, which have been reported to have a positive correlation with butyric acid production (Wang et al., 2018). Our results showed genera *Blautia*, *Anaerotruncus*, and *Oscillibacter* were all enriched in the WD group, which may all contribute to the higher acetate, butyrate, and total SCFA concentration in the cecum of broilers. On the other hand, broilers fed the WB diet decreased the abundance of *Oscillospira*, which of the genera have been reported to have a potential anti-inflammatory effect (Santoru et al., 2017). The anti-inflammatory effect of the WB diet has also been further confirmed by our experimental results of IL-6 mRNA level enhancing and jejunum occludin attenuating, which indicate an inflammatory response and concomitant intestinal barrier dysfunction. A previous study has reported that genera *Peptococcus* does not have carbohydrate fermentation functions (Liang et al., 2019). Wheat-containing diets have higher NSP content than corn-containing diets. Our results showed that genera *Peptococcus* was lower in the WD group than that in the BD group, which may reduce the degradation of NSP, thus decreasing the nutritional value and increasing the detrimental effects of NSP. Those negative effects may subsequently result in the poor growth performance of broilers fed WD diet. The *Bilophila* abundance is higher in the WED group than in the BD group, which may contribute to the BW of broilers as *Bilophila* was positively correlated with obesity in humans (Turnbaugh et al., 2009). However, other studies have reported that *Bilophila* is a pathobiont, which is related to the bile acid dysregulation in mice (Banerjee et al., 2016). Previous studies have reported that genera *Eubacterium_hallii_group* was butyrate producers (Bunesova et al., 2018), whereas, *Enterococcus* was opportunistic pathogenic bacteria (Huang et al., 2018). In the current study, supplementation of enzyme combined with probiotics improved *Eubacterium_hallii_group* abundance and reduced *Enterococcus* abundance, indicating an additional protective effect of inactivated *Lactobacillus* on broiler intestinal health. Diet supplemented with NSP enzymes alone or combined with inactivated *Lactobacillus* improved the intestinal bacterial communities, thus improving gut health, which may further contribute to the improvement of growth performance of broilers. In SCFA production, broilers fed wheat diet had higher concentrations of acetate and total SCFA compared to those provided the BD diet. A wheat diet supplemented with NSP enzyme or combined with probiotics improved propionate or butyrate production compared to the BD diet. McCafferty et al. (2019b) reported that broilers fed a wheat diet produced more butyrate compared to a corn diet, but no significant difference in acetate and total SCFA was observed. This is probably related to the higher abundance of *Eubacterium_hallii_group* and *unclassified_f__Lachnospiraceae*, the two SCFAs-producing genera (Bunesova et al., 2018; Hu et al., 2019) in WED and WEPD groups in our study. Increment of SCFA production, which can be used as an energy source for the improved growth performance of broilers.

## Conclusion

Wheat-based diet has negative effects on growth performance, indicated by lower ADG, BW, and higher F/G compared with a corn-based diet, whereas supplementing multi-enzymes alone or combined with inactivated *Lactobacillus* improved the growth performance of broilers. The added multi-enzymes inhibited the expression of TNF-α and combined with inactivated *Lactobacillus* supplementation further improved the mRNA expression of claudin-1, ZO-1, and mucin-2, indicating that the beneficial effect of inactivated *Lactobacillus* focus more on protecting gut health. Moreover, dietary enzyme supplementation alone or with probiotics could improve SCFA-producing bacteria (*Eubacterium_hallii_group* and *unclassified_f__Lachnospiraceae*) and further improve propionate and butyrate concentration. Collectively, our results suggested that the better growth performance might be related to an improvement in intestinal barrier function.

## Data availability statement

The data presented in this study are deposited in the NCBI Sequence Read Archive database, accession number: PRJNA825386.

## Ethics statement

The animal study was reviewed and approved by The Animal Ethics Committee of the Institute of Animal Sciences, Chinese Academy of Agricultural Sciences reviewed and approved the experimental protocol (Ethics Approval Code: IAS2021-233).

## Author contributions

QG, YW, and TM conceived and designed the experiments and wrote—review and editing. JL, GB, QG, and RZ performed the experiments. QG and YW analyzed the data. HZ, HP, and LL contributed to the reagents and materials. QG wrote—original draft. All authors read and approved the final manuscript.

## Footnotes

1
www.majorbio.com

2
https://www.microbiomeanalyst.ca/
