# Supplementary material for: Supplementation of multi-enzymes alone or combined with inactivated Lactobacillus benefits growth performance and gut microbiota in broilers fed wheat diets
Source: Front Microbiol. 2022 Aug 1;13:927932. doi: 10.3389/fmicb.2022.927932 (PMC9376439; doi:10.3389/fmicb.2022.927932)
Supplement: Supplementary file 1 [file Data_Sheet_1.docx]

**Figure S1**
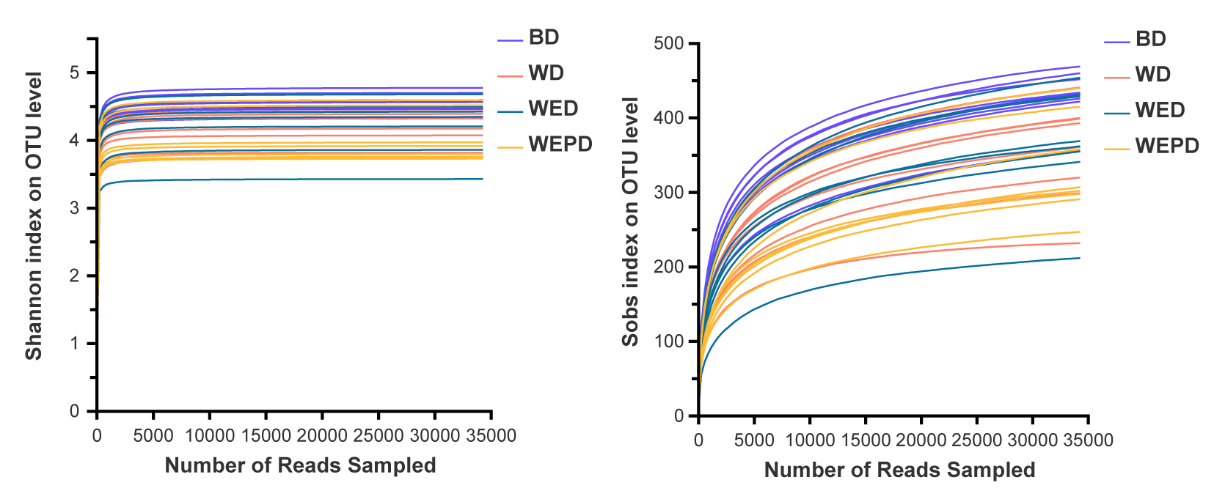
 Rarefaction curves of Sobs index and Shannon index on OTU level for each sample of cecal digesta.

**Table S1**. Primers for target genes.

| Gene | GenBank ID | Primer set (5′-3′) | Produce size, bp |
| --- | --- | --- | --- |
| β-actin | NM_205518.2 | F: CCAGCCATGTATGTAGCCATCCAG | 93 |
|  |  | R: GGTAACACCATCACCAGAGTCCATC |  |
| *ZO-1* | XM_046925214.1 | F: TCTTCCTCCTCCCGCTTCTTCAC | 81 |
|  |  | R: AGAGATGGTGGTGTAGGCAGTGG |  |
| Occludin | XM_046904540.1 | F: CGTCATGCTCATCGCCTCCATC | 126 |
|  |  | R: TTGAGGTAGGTGCTGCCGTAGG |  |
| Claudin-1 | NM_001013611.2 | F: GACCAGGTGAAGAAGATGCGGATG | 107 |
|  |  | R: CGAGCCACTCTGTTGCCATACC |  |
| Mucin-2 | XM_040673077.2 | F: GTCATTCCAAGGTGAACCCATCTCC | 105 |
|  |  | R: CAGCAACAGCAGAACAGAAGCAATC |  |
| IL-1β | XM_046931582.1 | F: GCTCTACATGTCGTGTGTGATGAG | 80 |
|  |  | R: TGTCGATGTCCCGCATGA |  |
| IL-6 | NM_204628.2 | F: GAAATCCCTCCTCGCCAATCTGAAG | 108 |
|  |  | R: GCCCTCACGGTCTTCTCCATAAAC |  |
| IL-10 | NM_001004414.4 | F: GCTGTCACCGCTTCTTCACCTG | 98 |
|  |  | R: GGCTTTGTAGATCCCGTTCTCATCC |  |
| TNF-α | XM_046927262.1 | F: CTGTTTCTGCCTCTGCCATC | 192 |
|  |  | R: GGGTTCATTCCCTTCCCATCT |  |

*ZO-1*, zonula occludens 1; IL-1β, Interleukin-1β; IL-6, Interleukin-6; IL-10, Interleukin-10; TNF-α, tumor necrosis factor-α
